# From Survival to Recovery: Understanding the Life Impact of an Acute Aortic Dissection Through Activity, Sleep, and Quality of Life

**DOI:** 10.3390/jcm14030859

**Published:** 2025-01-28

**Authors:** Nora Bacour, Simran Grewal, Rutger T. Theijsse, Robert J. M. Klautz, Nimrat Grewal

**Affiliations:** 1 Department of Cardiothoracic Surgery, Amsterdam University Medical Center Location AMC, 1105 AZ Amsterdam, The Netherlands; n.bacour@amsterdamumc.nl (N.B.); r.t.theijse@amsterdamumc.nl (R.T.T.); r.klautz@amsterdamumc.nl (R.J.M.K.); 2 Department of Orthopaedic Surgery, Onze Lieve Vrouwe Gasthuis, 1091 AC Amsterdam, The Netherlands; s.k.grewal@olvg.nl; 3 Department of Cardiothoracic Surgery, Leiden University Medical Center, 2333 ZA Leiden, The Netherlands; 4 Department of Anatomy and Embryology, Leiden University Medical Center, 2333 ZA Leiden, The Netherlands

**Keywords:** acute aortic syndrome, aortic dissection, quality of life, activity, survivors

## Abstract

**Background/Objectives:** An acute aortic dissection (AAD) is a cardiovascular emergency with high mortality rates if left untreated. Survival has increased due to improvements in diagnosis and therapy. However, during their recovery, survivors frequently encounter major social, psychological, and physical challenges. This study aimed to evaluate the recovery experience of AAD survivors in The Netherlands. Insights on sleep quality, physical activity, and quality of life were collected from a unique nationwide cohort of AAD survivors recruited through the national patient support network ‘Stichting Aorta Dissectie Nederland’. **Methods:** This study was conducted among AAD survivors who were recruited through a national association for aortic dissection known as ‘Stichting Aorta Dissectie Nederland (SADN)’. The participants (n = 61) completed questionnaires assessing demographic data, physical activity, sleep quality, and health-related QoL. **Results:** The cohort had a mean age of 60.1 years, and 47.5% of the participants were female. The prevalence of sleep disruptions was high, as 55.7% of the people were categorized as bad sleepers (PSQI > 5). Poor sleep was associated with low physical activity and a higher BMI. The physical activity levels varied, with 47.5% reporting moderate activity levels and 44.3% reporting high activity levels. The QoL scores varied greatly among the participants, with significant impairment across all fields and reduced enthusiasm for daily activities. Poor sleepers reported significantly lower QoL (*p* < 0.001). **Conclusions:** Our study highlights significant gaps in post-AAD care, particularly addressing QoL, sleep, and physical activity. By acknowledging the multifaceted nature of recovery, healthcare providers can develop tailored interventions that empower survivors to achieve better quality of life.

## 1. Introduction

An acute aortic dissection (AAD) is a life-threatening cardiovascular emergency characterized by the separation of the layers of the aortic wall, which is triggered by a tear in the intimal vascular layer. If untreated, an AAD is rapidly fatal, with an estimated mortality rate of 40% upon initial presentation. Mortality rates soar by 1% per hour from symptom onset, reaching as high as 90% annually [1]. However, advances in disease awareness, diagnostic tools, and therapeutic interventions have significantly improved survival rates for AAD patients, with over 80% of patients now surviving the initial event [2,3].

Despite this progress, the sudden and life-altering nature of AAD forces survivors to grapple with significant challenges that extend far beyond the acute phase. For AAD survivors, the path from survival to recovery is plagued by physical, emotional, and social obstacles. Survivors report diminished quality of life (QoL), psychological distress, and physical impairment, alongside an increased burden of managing long-term post-dissection complications [2]. Uncertainty about the disease and rehabilitation further compound the recovery process, which underscores the need for comprehensive post-discharge care [4]. Current European and American guidelines recommend structured cardiac rehabilitation with controlled physical activity and disease surveillance [5,6,7]. However, such programs often overlook the broader, subjective recovery experience of survivors.

While much of the existing literature on AAD mainly focuses on improving survival outcomes, limited attention has been given to the recovery phase and the impact of an AAD on the daily routines and quality of life of patients. Sleep patterns, physical activity, and quality of life are pivotal but understudied domains that could potentially influence the recovery of AAD survivors. Sleep disturbances have been frequently reported after cardiovascular events [8]. Following surgical intervention, sleep disturbances have even been associated with increased cardiovascular risks, heightened pain perception, reduced quality of life, and impaired healing [9]. Physical inactivity is also a well-documented risk factor for both the development of cardiovascular morbidity and delayed recovery from cardiovascular events, yet many AAD survivors struggle to re-engage in physical activity due to fear or a lack of guidance [10,11,12,13].

We aimed to fill these knowledge gaps by studying the recovery experience of AAD survivors in The Netherlands. Insights on sleep quality, physical activity, and quality of life were collected from a unique nationwide cohort of AAD survivors recruited through the national patient support network ‘Stichting Aorta Dissectie Nederland’. A comprehensive understanding of the post-dissection health status of AAD survivors will inform the development of targeted interventions and support systems, ultimately improving the long-term outcomes and well-being of AAD survivors.

## 2. Materials and Methods

### 2.1. Ethical Considerations

This study was conducted in adherence to the ethical principles outlined in the Declaration of Helsinki and the Good Clinical Practice guidelines. The confidentiality and anonymity of all collected data were ensured through these principles. The study protocol was examined and approved by the Medical Ethical Committees of Leiden University Medical Center. It was covered under the biobank protocol and was assigned the following reference number: B21.051/MS/ms. Written informed consent was acquired from all participants.

### 2.2. Patient Selection

This study was conducted among AAD survivors who were recruited through a national association for aortic dissection known as ‘Stichting Aorta Dissectie Nederland (SADN)’. The participants were reached via a national aortic dissection awareness event and the website of the national association for aortic dissections (‘SADN’). Individuals who expressed interest in this study provided consent and were invited to complete questionnaires designed to provide insights into their recovery experiences.

### 2.3. Questionnaire

The questionnaire collected demographic variables including age, sex, weight, height, blood pressure, type of AAD (A, B, both, or unknown), and the presence of risk factors such as smoking. To complement these demographic data, we conducted a comprehensive assessment of QoL, physical activity levels, and sleep quality using established and validated instruments.

Physical activity was measured using the International Physical Activity Questionnaire (IPAQ) [14], which categorizes patients’ activity levels as low, moderate, or high based on standardized scoring criteria, as described by Cheng et al. [15].

Health-related QoL was evaluated by assigning scores from 0 (no problems) to 4 (the complete inability to perform a function) across domains such as mobility, self-care, usual activities, pain, and anxiety [16]. The scores were evaluated individually and compared to findings from a neighboring study [17], as individual Dutch scores were unavailable. Overall QoL was assessed using the EQ-index. This index uses a Dutch value set to score individual scores. These combined results form a score from −1 to 1, where 0 is the threshold for a QoL better or worse than being dead [18].

Sleep quality was assessed using the Pittsburgh Sleep Quality Index (PSQI) [19], a reliable instrument that measures sleep duration, efficiency, latency, and disturbances. When scoring the PSQI, seven component scores are derived, each scored from 0 (no difficulty) to 3 (severe difficulty). The individual components of the PSQI include sleep quality, sleep latency, sleep duration, sleep efficiency, sleep disturbances, the use of sleeping medication, and daytime dysfunction. These component scores are summed to generate a global score, which ranges from 0 to 21. Higher global scores reflect poorer sleep quality, with a score above 5 serving as the threshold for poor sleep.

Finally, key variables such as smoking rates, hypertension, and obesity were analyzed to provide additional insights.

### 2.4. Statistical Analyses

To summarize the baseline characteristics of the post-dissection patients, descriptive statistics were employed. Variables were assessed for skewness and compared using various statistical tests. To compare two unpaired groups, an unpaired *t*-test was used for normally distributed data, while a Mann–Whitney U test was applied for non-normally distributed data. To compare three or more unpaired groups, a one-way Anova test was used was used for normally distributed data, while a Kruskal Wallis test was applied for non-normally distributed data. Categorical data were categorized into ordinal data, assessed using a chi-square test for trend, and binary data, assessed using a chi-square test or Fisher’s exact test if the assumption of sufficient cell counts was not met. A *p*-value < 0.05 was considered significant. All statistical analyses were conducted using IBM SPSS version 27.0.

## 3. Results

### 3.1. Baseline Demographics

The study population consisted of 61 participants with a mean age of 60.1 years (SD: ±9.5). Of these, 47.5% were female and 52.5% were male. The participants had an average height of 177.9 cm (SD: ±9.9) and an average weight of 85.7 kg (SD: ±16.7), corresponding to a mean BMI of 27.1 kg/m2 (SD: ±4.8). The average reported blood pressure was 123/77 mmHg. Regarding smoking habits, only 3.3% were current smokers, while 37.7% were former smokers.

In terms of the type of dissection, 47.5% of the participants had a Type A dissection, 23% had a Type B dissection, and 19.7% had both. Most patients (82%) underwent surgical treatment, while 18% received conservative management. The patients reported an average health scale score of 71.4 out of 100 (SD: ±14.7). The average sleep duration was 7.3 h (SD: ±1.3) (Table 1).

### 3.2. Physical Activity Levels

Based on the IPAQ survey [15], 47.5% of the participants were categorized as moderately active, while 44.3% were classified as highly active. On average, the participants reported walking 5.9 days per week, with each walking session lasting 76 min. The mean daily step count was 6776. Moderate activity, defined as engaging in physical activity equivalent to at least 30 min of moderate-intensity exercise on most days [20], was performed 3.7 days per week, on average, with each session lasting 90 min. Vigorous exercise, defined as physical activity equivalent to at least one hour per day of moderate- to high-intensity activity [20], was performed 1.3 days a week, with an average duration of 77 min per session. Despite these activity levels, the participants reported an average sedentary time of 8 h and 5 min per day.

### 3.3. Sleep Outcomes

Sleep quality was assessed by the global PSQI score and individual PSQI components (Table 2). Based on the global PSQI score, 55.7% of the study participants were classified as poor sleepers (global PSQI scores > 5), while 44.3% were good sleepers (global PSQI scores ≤ 5). The average PSQI score was 6.1 (SD: ±3.5).

Low activity levels were significantly associated with worse sleep quality, with the participants in this group reporting a mean PSQI score of 11.2 compared to 5.7 for those with moderate or high activity levels (*p* < 0.001).

Additionally, the overweight participants (BMI > 25) exhibited higher PSQI scores (6.8 vs. 5.0; *p* = 0.46). Approximately, 20% of the patients reported moderate to severe sleep problems.

Table 2 shows the average PSQI (sub-) scores in our group of post-dissection patients. The sub-scores range from 0 (no difficulty) to 3 (severe difficulty), while the global score ranges from 0 to 21. Higher global scores indicate poorer sleep quality, with a score above five serving as the threshold for poor sleep. You can see that, on average, the patients slept poorly. The sub-scores also reveal significant deficiencies in achieving qualitatively good sleep. Given that sleep has been shown to be critically important for QoL and recovery post-surgery, the current lack of good sleep and the importance of providing effective support for better sleep are highlighted.

### 3.4. Quality of Life

**Figure 1:**
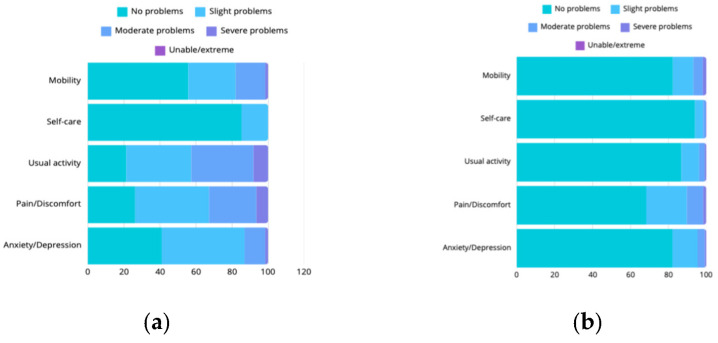
Quality of life assessment. This figure compares the quality of life assessment within our study population (**a**) with average EQ-5D ratings (**b**) [17]. You can see that compared to an average population in a nearby Western country, significantly more complaints were reported in our study population. This underscores the importance of implementing better personalized management strategies in post-dissection patients to improve health-related QoL.

Health-related QoL was assessed across the domains of mobility, self-care, usual activities, pain/discomfort, and anxiety/depression. The reported QoL scores varied greatly among the participants. Compared to the general population of another Western country (Germany [21]), the AAD survivors reported significantly higher rates of moderate or greater problems across all dimensions (21.6% vs. 5.5%) (Figure 1). The overall QoL, as assessed by the EQ-Index, scored 0.7 (SD: ±0.2), which was lower compared to the average of 0.9 reported in the Dutch value set [18].

Notably, 42.62% of the participants reported moderate or severe problems with enthusiasm for daily activities. Overall QoL ratings, with lower scores indicating worse QoL, showed that poor sleepers reported significantly lower QoL compared to good sleepers (0.7 vs. 0.8, *p* = 0.016). Interestingly, no statistical association was identified between the overall QoL scores and activity levels (*p* = 0.895).

## 4. Discussion

In this study, we aimed to provide a comprehensive understanding of the recovery journey of AAD survivors, focusing on critical aspects such as QoL, physical activity levels, and sleep, which are often underexplored in the existing literature.

### 4.1. Physical Activity Levels

This study revealed that most AAD survivors are moderately active during their recovery. While evidence from previous research suggests that moderate-intensity cardiovascular activity is cardioprotective [22], the findings from our study should be interpreted with caution. The relatively high activity levels among our participants may have partly resulted from self-selection bias, as individuals with the motivation for behavioral change tend to engage in research studies. Given that the participants may have been intrinsically motivated when participating in our study, it is not surprising that most of them reported adequate levels of activity.

Interestingly, e-health interventions could serve as practical tools to bridge the gap between motivation and sustainable behavioral change. Digital platforms can offer tailored activity plans and real-time (neuro)feedback while addressing barriers such as fear of overexertion or a lack of guidance by healthcare professionals [23]. Given the aging demographic of AAD survivors, further research is warranted to evaluate the long-term impacts of such interventions on physical activity and overall recovery.

### 4.2. Sleep Outcomes

Sleep disturbances were highly prevalent in our study, with over half of the participants classified as poor sleepers. These findings align with previous research that links poor sleep quality with worse cardiovascular recovery [24]. Notably, the participants with lower activity levels or a BMI greater than 25 reported worse PSQI scores, indicating that physical activity and weight management could indirectly influence sleep quality.

The observed association between sleeping difficulties, as indicated by individual PSQI scores, and the QoL assessment suggests that addressing sleep disturbances in post-AAD care could improve patients’ QoL and thus their overall recovery.

The implications of poor sleep extend beyond QoL. Sleep disorders have been linked to increased cardiovascular events, prolonged recovery times, and greater pain catastrophizing [9]. This highlights an urgent need to incorporate strategies to improve sleep hygiene into post-dissection care plans. Strategies such as cognitive behavioral therapy for insomnia, relaxation techniques, and wearable sleep monitors could offer accessible solutions for the AAD population [25,26].

### 4.3. Quality of Life

The substantial reduction in QoL among the AAD survivors compared to a normative population underscores the multidimensional impact of this condition. Anxiety, pain, and reduced enthusiasm for daily activities were among the most frequently reported challenges among the participants. Importantly, both walking and good sleeping habits emerged as protective factors, emphasizing the interplay between physical and mental health.

To improve QoL among AAD survivors, a more integrated rehabilitation approach is essential. Severe physical activity restrictions, for instance, have been shown to reduce QoL in patients diagnosed with an AAD [22]. We encourage a holistic treatment plan with, in addition to traditional physical therapy, interventions that should also address psychological well-being through peer support groups or counseling. These elements could help AAD survivors to rebuild their confidence and regain a sense of normalcy in their daily lives.

### 4.4. Limitations and Future Directions

While our study offers valuable insights by addressing patients’ post-dissection experiences, it has several limitations. Besides studying overall QoL, our study compared individual QoL parameters to those from a descriptive German study, which may not have adequately reflected the Dutch population. However, this paved the way for future research to compare QoL ratings from baseline studies or to define EQ-5D-5L values specific to The Netherlands.

The cross-sectional design did not account for changes over time, limiting our ability to establish causal relationships. Another limitation was the reliance on voluntary participation, which may have skewed the sample towards more health-conscious individuals, potentially underestimating the challenges faced by less motivated or proactive survivors.

Future research should adopt longitudinal methodologies, tracking patients from hospital discharge to several years post-recovery. This would enable a more nuanced understanding of how activity levels, sleep, and QoL evolve over time. Additionally, exploring the roles of socio-economic factors and healthcare accessibility could provide a more holistic view of recovery disparities.

## 5. Conclusions

Our study highlights significant gaps in post-AAD care, particularly addressing QoL, sleep, and physical activity. A holistic recovery program incorporating personalized activity guidelines, sleep hygiene strategies, and mental health support is crucial to improve overall well-being and outcomes. By acknowledging the multifaceted nature of recovery, healthcare providers can develop tailored interventions that empower survivors to achieve better quality of life.

## Figures and Tables

**Table 1:** Group description.

	N (61)	Mean	Std. Deviation
Age	60	60.1	9.5
Sex	29 (47.5%) Female 32 (52.5%) Male		
Weight (kg)	61	85.7	16.7
Height (cm)	60	177.9	9.9
BMI	60	27.1	4.8
Blood pressure	58 (systolic) 51 (diastolic)	123/77	
Type of dissection			
Type A dissection	29 (47.5%)		
Type B dissection	14 (23%)		
Both	12 (19.7%)		
Missing data	6 (9.8%)		
Surgical intervention	50 (82%)		
Smokers	2 current (3.3%) 23 ex-smokers (37.7%) 35 non-smokers (57.4%)		
Health scale rating	60	71.4	14.6

**Table 2:** PSQI dimensions.

Categories	Mean ± SD	Median (IQR)	Min~Max
Global PSQI scores (total score)	6.1 ± 3.5	6.0 (5.0)	0~16
Component 1: Subjective sleep quality	1.1 ± 0.7	1.0 (0.0)	0~3
Component 2: Sleep latency	1.2 ± 1.1	1.0 (2.0)	0~3
Component 3: Sleep duration	0.3 ± 0.7	0.0 (0.0)	0~3
Component 4: Habitual sleep efficacy	0.8 ± 1.1	0.0 (1.0)	0~3
Component 5: Sleep disturbances	1.5 ± 0.5	1.0 (1.0)	1~3
Component 6: Use of sleeping medications	0.3 ± 0.8	0.0 (0.0)	0~3
Component 7: Daytime dysfunction	1.0 ± 0.8	1.0 (0.50)	0~3

PSQI, Pittsburgh Sleep Quality Index; SD, Standard Deviation; IQR, Interquartile Range.

## Data Availability

The data presented in this study are available on request from the corresponding author.

## Abbreviations

The following abbreviations are used in this manuscript:

AAD	acute aortic dissection
QoL	quality of life
SADN	Stichting Aorta Dissectie Nederland (Aortic Dissection Association Netherlands)
